# Explaining the intention and behaviours of interinstitutional collaboration in chronic disease management among health care personnel: a cross-sectional study from Fujian Province, China

**DOI:** 10.1186/s12913-023-09453-0

**Published:** 2023-05-11

**Authors:** Li Teng, Yue Dai, Tao Peng, Yuan Su, Lingyi Pan, Yueping Li

**Affiliations:** 1 The School of Public Health, Fujian Medical University, Fuzhou, China; 2 The School of Management, North Sichuan Medical College, Nanchong, China; 3 The School of Basic Medicine, North Sichuan Medical College, Nanchong, China; 4 The School of Arts and Sciences, Fujian Medical University, No. 1, Xueyuan Road, Shangjie Town, Minhou County, Fuzhou, China

**Keywords:** Theory of planned behaviour, Chronic disease management, Interinstitutional collaboration

## Abstract

**Background:**

The increasing number of chronic diseases consumes a large amount of health resources and puts a huge burden on health service system. The integrated management of chronic diseases in Sanming City aims to improve the efficiency and quality of chronic disease management through the collaboration between different levels of medical institutions.

**Aim:**

The aim of the present study was to use the theory of planned behaviour (TPB) to examine the intention and behaviours of interinstitutional collaboration in chronic disease management (ICCDM) among healthcare personnel.

**Methods:**

A cross-sectional study of 274 health care personnel was conducted in medical institutions in Fujian Province, China, from March 2022 to April 2022. A self-administered questionnaire based on TPB theory was applied to measure the participants’ ICCDM behaviours.

**Results:**

The proposed TPB model revealed that attitude was significantly and positively associated with behaviour intention, and behaviour intention and perceived behavioural control were significant predictors of ICCDM behaviour.

**Conclusion:**

TPB provides insights into ICCDM behaviour. Due to the fact that attitude, perceived behavioural control, and behavioural intention towards ICCDM behaviour were demonstrated to be significant predictors of ICCDM behaviour, these factors may be a promising focus of ICCDM interventions in the integrated management of chronic diseases in China.

**Supplementary Information:**

The online version contains supplementary material available at 10.1186/s12913-023-09453-0.

## Introduction

### Background

Chronic diseases are the number one threat to human health. According to data released by the World Health Organization in 2018, an estimated 41 million people died from non-communicable diseases globally in 2016, accounting for 71% of total deaths (57 million) [1]. This poses huge challenges for health service systems, especially in developing countries. The increasing number of chronic diseases consumes a large amount of health resources and puts a huge burden on health service system and government finance. In this context, the concept of integrated health service comes into being, aim of improving the overall service efficiency and service quality by adjusting the combination of health system and the allocation of health resources, etc., while not increasing or less the total health investment.

As for China, the main aspect of chronic disease management in China was the primary health care institutions represented by township health centres and community health centres [2]. However, due to the unbalanced allocation of health resources, China’s primary health care institutions lack service capacity, and the effect of chronic disease management does not meet expectations [3, 4]. Moreover, the lack of system integration between different levels of medical institutions in China makes it difficult to provide continuous disease management services. The traditional community chronic disease health management model has made it difficult to cope with the new health environment and is in urgent need of guidance and assistance from superior hospitals [5].

In 2016, based on the fact that the continuity of health services in China is poor, the Chinese government, the World Bank, and the World Health Organization issued a joint research report and proposed the construction of “people-centred integrated health care (PCIC)” ([6, 7]. The purpose of integrated health services is to integrate health resources for the improvement of the efficiency of health services and for providing continuous and systematic health services for residents as much as possible [8]. For chronic disease management, to ensure efficiency and quality, it is necessary for clinical and public health professionals to cooperate with each other as well as with health departments and institutions at different levels to facilitate the seamless connection of all service links and to ensure that the entire process from disease occurrence to management is fully linked to patients. A recent study from China found that well-integrated health care delivery systems can improve the effectiveness of multidisciplinary team-based chronic disease management, both in terms of improved patient health outcomes and savings in health care costs [9]. The integration of the health service system is conducive to the realization of continuous chronic disease management, which is an important direction of China’s current medical reform [10, 11].

### Sanming model-integrated chronic disease management

Sanming City in Fujian Province is famous nationwide for its health care reform [12], and its efforts to integrate health services have become a model for other regions. The goal of the latest reform is to change the mode of medical service from “disease-centred” to “health-centred” in the context of the integration of the health service system. To achieve this goal, cooperation between hospitals and primary medical institutions for conducting chronic disease management has become a focus.

**Fig. 1:**
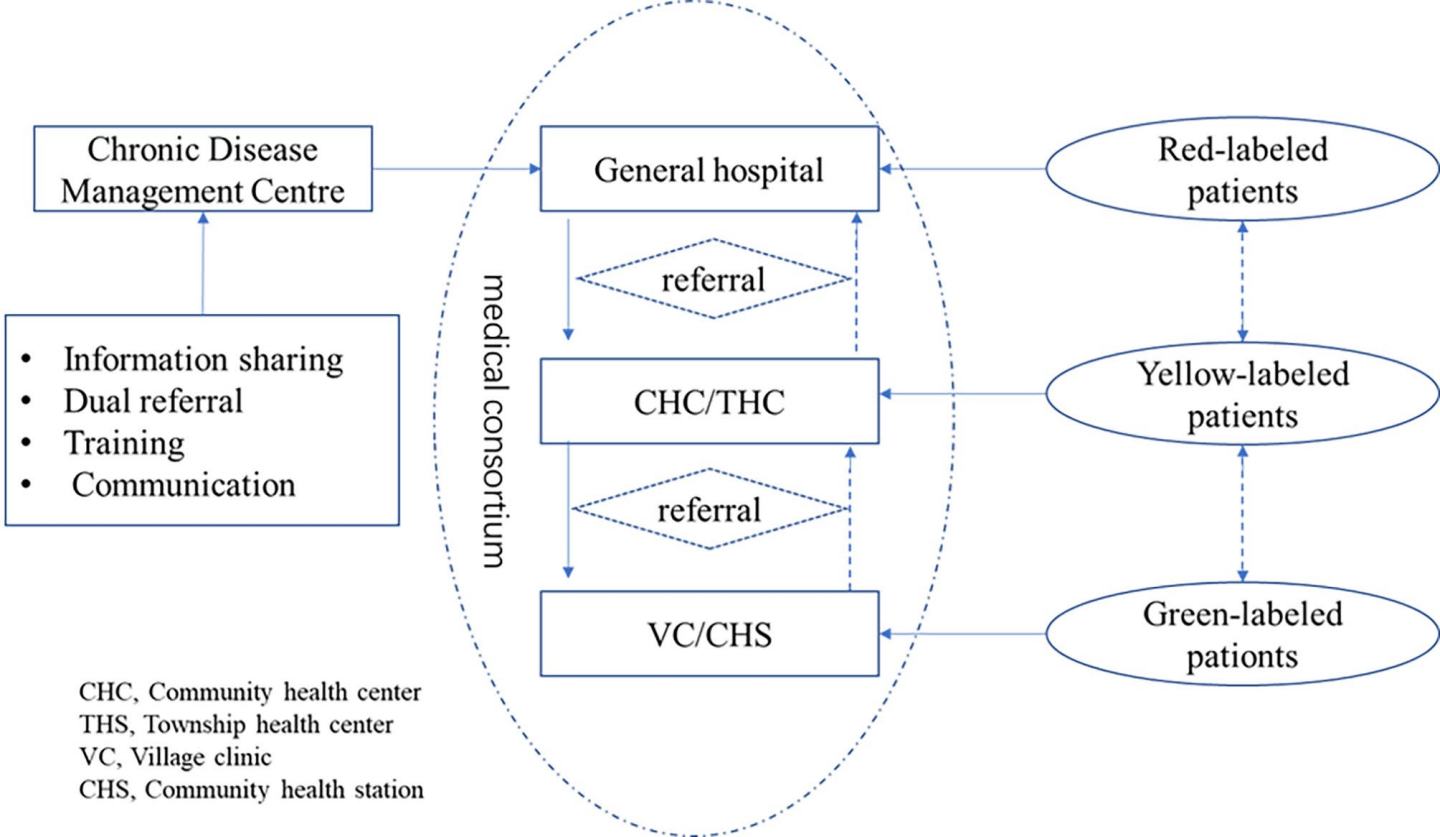
Integrated management of chronic diseases in Sanming City

The Sanming model is known as “integrated chronic disease management”. This represents a chronic disease management model based on Medical Alliance. Under the leadership of the General Hospital, with community health service centres and township health centres as the main force, the chronic disease management team is formed in conjunction with village clinics/community health service stations to provide integrated management services of “Grading, Classification and Labelling” for patients with chronic diseases. Grading refers to the establishment of chronic disease management networks at the county, township and village levels. Classification refers to the establishment of chronic disease management centres in general hospitals according to disease types. Furthermore, labelling refers to the classification of patients into green, yellow and red markers according to the severity of their disease. Specifically, village clinic and community health service stations are responsible for green-labelled patients, township health centres and community health service centres are responsible for yellow-labelled patients and general hospitals are responsible for red-labelled patients. It is worth noting that the colours of the labels have been dynamically adjusted according to the changes in the patient’s condition. See Fig. 1 for the specific service flow and pattern.

### Research question

The Sanming model is emerging in the context of the vertical integration of the health system. The integration of the health system is not unique to China; in countries with a high degree of marketization, collaboration between medical institutions is mainly voluntarily formed [13, 14]. However, the bulk of China’s health system is represented by public institutions. Therefore, most medical alliances in China are based on administrative divisions and form cooperative relations through government orders. This aspect raises a series of fresh questions, such as the degree of interinstitutional collaboration in this type of administratively mandated integration, if close collaboration can be formed among health care personnel and which factors affect health care personnel collaborations.

The aim of the present study was to use the theory of planned behaviour (TPB) to examine the intention and behaviours of interinstitutional collaboration in chronic disease management (ICCDM) among health care personnel. Based on the TPB, the present study tested a model including attitude, subjective norms, perceived behavioural control and intention as predictors of ICCDM behaviour among Sanming health care personnel through a questionnaire survey.

## Methods

### Theoretical framework and research hypothesis

The TPB was proposed by Ajzen in 1990 and is predominantly used to understand and predict human intentions and behaviours[15]. Ajzen believed that behavioural intention is an essential process of behavioural expression; thus, one’s behavioural intention can determine behaviour to a large extent under sufficient conditions. Behavioural intention is affected by attitude, subjective norms and perceived behavioural control. In addition, perceived behavioural control can also affect behaviour choices [16].

The TPB has been widely applied in numerous studies, including behavioural changes among physicians [17, 18]. This study suggests that TPB can provide a theoretical framework for the behavioural changes of health care personnel involved in chronic disease management. Attitudes refer to one’s evaluations of ICCDM behaviours (e.g., ICCDM leads to a better quality of care for patients); subjective norms refer to the social pressure that an individual feels when performing ICCDM behaviours (e.g., leadership attaches importance to ICCDM); perceived behavioural control refers to an individual’s perception of how difficult it is to perform ICCDM behaviours (e.g., “I have enough time and energy to participate in ICCDM.”). Given the above, the following hypotheses were examined in this study.

H1: Health care personnel’s attitudes towards ICCDM positively impact their intention to participate in ICCDM.

H2: Health care personnel’s subjective norms positively impacted their intention to participate in ICCDM.

H3: Health care personnel’s perceived behavioural control positively impacts their intention to participate in ICCDM.

H4: Health care personnel’s intention to participate in ICCDM positively impacts their ICCDM behaviour.

H5: Health care personnel’s perceived behavioural control positively impacts their ICCDM behaviour.

### Participants, design and procedure

A cross-sectional study was conducted in Sanming City. In this study, two urban areas and two rural counties of Sanming City were selected by using random cluster sampling. Each district (county) has established its own medical alliance, and all four medical alliances were investigated from March 21, 2022, to April 20, 2022. Due to the fact that the study took place during the outbreak of COVID-19, an online survey was conducted to reduce social contact. Electronic questionnaires and instructions were distributed to medical personnel who were involved in chronic disease management via general hospitals. A total of 383 personnel were eligible for this study. After excluding incomplete responses and invalid responses (same answers to all questions), a total of 274 eligible questionnaires were collected thus resulting in a response rate of 71.5%.

Those individuals interested in participating in the study were required to provide informed consent before completing the online survey. Ethical approval was obtained (Approval: FJMU 2022 NO.88) prior to data collection.

### Measures

This study designed a questionnaire based on five basic constructs from the TPB. According to standardized guidelines[19], all of the items were drawn from previous studies or developed in consultation with experts. The list and references of items in this study is presented in Supplementary Material S1. Before the formal investigation, we conducted a preliminary survey and preliminarily evaluated the reliability and validity of the questionnaire. Participants also completed a brief demographic information survey.

#### Primary outcome: collaborative Behaviour (CB, 3 items)

The first item measured the number of times participants conducted ICCDM over the last three months. Participants were presented with an introduction about ICCDM, which elaborated on the specific form of ICCDM. The options were divided into five levels: none, 1–3, 4–6, 7–9 and more than 10 times.

The second item aimed to assess the frequency of continuous service delivery via the following question: “Your experience working with members from other institutions to provide services to the same patient?”. The third item measured the frequency of referrals via the following question: “In the past year, have you referred any patients with chronic diseases to a cooperative organization?”. Consistent with the rest of the questionnaire, these two questions were answered at five levels, ranging from “never” to “always”.

#### TPB constructs

Based on the TPB model, our self-administered questionnaire assessed participants’ collaborative attitudes (CA), subjective norms (SN), perceived behavioural control (PBC) and collaborative intention (CI) about chronic disease management. The questionnaire was intuitively measured by using a Likert-5 scale. A higher score represented a higher cognitive level (Supplementary Material S1).

#### Demographic variables

Participants self-reported their age, gender, facility setting, occupational identity, education level and the years of service.

### Data analysis

The statistical package for social science for Windows (SPSS, version 22.0; Armonk, NY) and AMOS (version 24.0) were jointly used to conduct the statistical analyses. Prior to establishing the participants’ behaviour model, the reliability and validity of the TPB survey were determined by calculating Cronbach’s alpha and a factor analysis. Based on the theoretical framework of TPB, a structural equation model was used to understand the correlation of ICCDM behaviour and its determinants.

Goodness-of-fit indices were applied to evaluate the fit of the structural equation model, including the Chi squared freedom ratio (1 < χ2/df < 3), root mean square error of approximation (RMSEA < 0.08), the comparative fit index (CFI > 0.90), Tucker–Lewis index (TLI > 0.90) and the incremental fit index (IFI > 0.90).

## Results

### Participants

As shown in Table 1, the majority of the participants were female (65.59%). The median age of the participants was 41 years, and the median working years was 15.5 years. The education level was mainly university degree (68.98%), followed by high school and below (29.93%) and graduate (1.09%) degrees. The occupational identities included doctors (37.96%), nurses (27.74%), public health workers (14.60%), medical technicians (11.32%) and others (8.39%). In addition, the participants came from hospitals (26.28%), township health centres (21.90%), village clinics (27.74%), community health service centres (21.53%) and community health service stations (2.19%), thus encompassing all levels of medical institutions.

**Table 1:** Demographic and work characteristics of the medical personnel

Characteristics	N (%)/median (IQR)
Gender	
Male	94 (34.31)
Female	180 (65.69)
Age	41.0 (31.0–51.0)
Facility setting*	
Village clinic	76 (27.74)
Community health station	6 (2.19)
Community health center	59 (21.53)
Township health center	60 (21.90)
Hospital	72 (26.28)
Occupational identity	
Nurse	76 (27.74)
Physician	104 (37.96)
Public health personnel	40 (14.60)
Medical Technician	31 (11.32)
Others	23 (8.39)
Education level	
Senior high school and below	82 (29.93)
University degree	189 (68.98)
Graduate	3 (1.09)
Length of service	15.5 (7.0-28.3)

IQR, interquartile range (25th to 75th percentile). *There were missing data

.

### Measurement score of participants’ perception and ICCDM behaviour

As seen from Table 2, the participants demonstrated a strong intention to engage in ICCDM (mean = 4.07, SD = 0.72). They also demonstrated a positive collaborative attitude (mean = 4.17, SD = 0.68), and they experienced high social pressure (subjective norms) of engaging in ICCDM (mean = 4.14, SD = 0.67). In contrast, the participants reported less frequent collaborative behaviour (mean = 2.61, SD = 1.07), and their perception of behavioural control of engaging in ICCDM was relatively negative (mean = 3.87, SD = 0.74).

**Table 2:** Descriptive statistics (correlations, square roots of AVE, means, standard deviations and Cronbach’s α coefficients) for all of the study constructs

	CA	SN	PBC	CI	CB	Mean	SD	Cronbach’s α
CA	0.849					4.169	0.682	0.928
SN	0.843	0.870				4.140	0.666	0.903
PBC	0.616	0.768	0.828			3.866	0.736	0.900
CI	0.848	0.714	0.518	0.885		4.064	0.721	0.880
CB	0.289	0.251	0.336	0.355	0.597	2.609	1.070	0.622

SD, standard deviation

### Preliminary analyses

A preliminary analysis was conducted to examine the relationship among all of the constructs, as well as the reliability and validity. Correlations and reliability can be found in Table 2. In the reliability analysis, the Cronbach’s α coefficient of the overall reliability of the questionnaire was 0.923, and all of the coefficients of the potential constructs were greater than 0.700, except for CB, which was close to 0.700. In the validity analysis, the overall KMO value and SIG value of the questionnaire were 0.933 and 0.000, respectively. KMO and Bartlett sphericity tests showed that the questionnaire had structural validity and could be used for the factor analysis. The square root AVE (see the diagonals in Table 2) of each potential construct exceeded the correlation between the construct and other constructs, thus indicating support for discriminant validity.

### Structural equation models

#### Model fit

According to the fitness test results shown in Table 3, the structural equation model had an excellent fit to these data (χ2/df = 2.648, IFI = 0.938, CFI = 0.937, TLI = 0.926 and RMSEA = 0.078).

**Table 3:** Fitting index of the model

Index	*χ* ^*2*^ */df*	*IFI*	*CFI*	*TLI*	*RMSEA*
Criterion	1–3	> 0.90	> 0.90	> 0.90	< 0.08
Fitting result	2.648	0.938	0.937	0.926	0.078

#### Path analysis of structural equation models

**Fig. 2:**
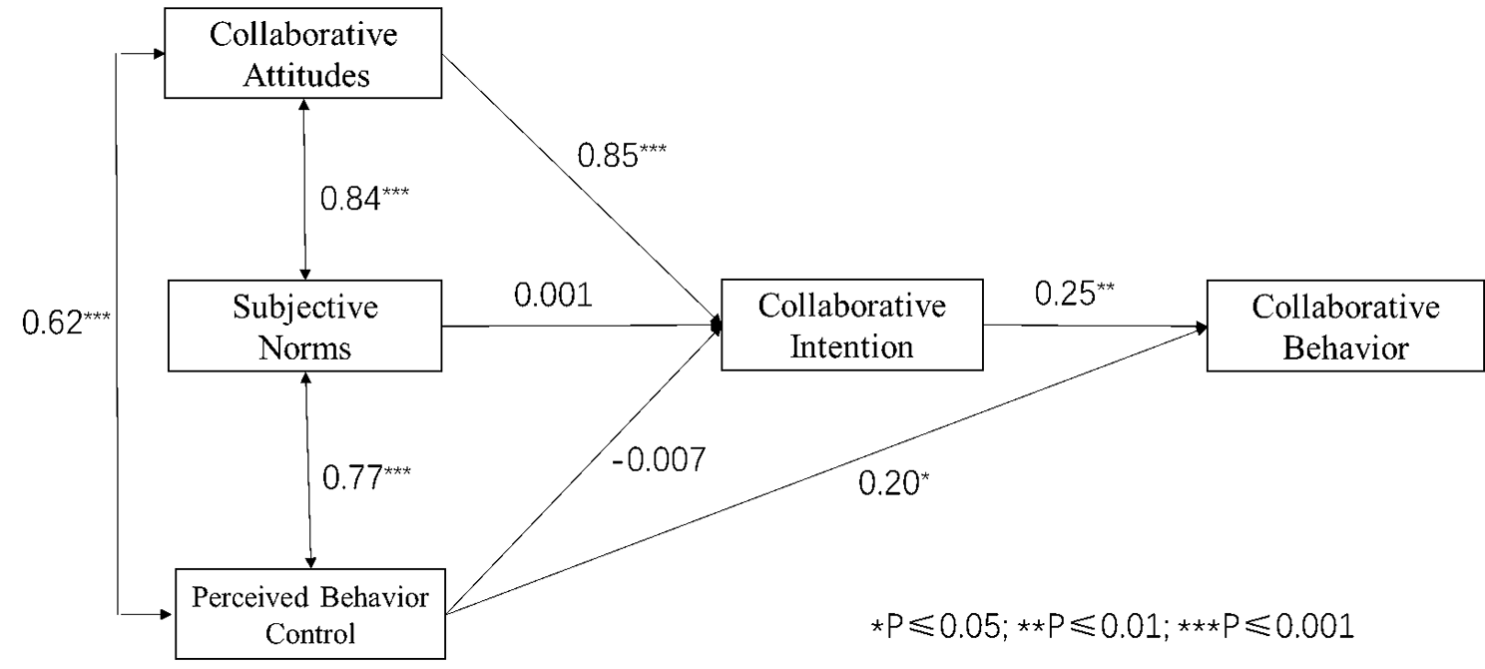
Path analysis of ICCDM under the theory of planned behaviour

Figure 2 shows the path test results of the TPB model, and Table 4 shows the verification results of the hypotheses that were proposed in this study. Attitudes (β = 0.85, P < 0.001) had significant positive effects on collaborative intention, thus indicating that H1 passed the verification test. However, subjective norms and perceived behavioural control had no significant impact on collaborative intention; therefore, H2 and H3 failed to pass verification. In addition, collaborative intention (β = 0.25, P < 0.01) and perceived behavioural control (β = 0.20, P < 0.05) had significant positive effects on collaborative behaviour, thus indicating that H4 and H5 were verified.

**Table 4:** Results of hypothesis examination

Hypothesis	Path	Standardized coefficient β	P value	Results
H1	CA→CI	0.850	***	Support
H2	SN→CI	0.001	0.993	Reject
H3	PBC→CI	-0.007	0.926	Reject
H4	CI→CB	0.249	**	Support
H5	PBC→CB	0.199	*	Support

*P ≤ 0.05; **P ≤ 0.01; ***P ≤ 0.001

## Discussion

Our study served as one of the first series of studies to explore the influencing factors of health care personnel’s ICCDM behaviour based on TPB theory. According to the well-fitting structural equation model, our study revealed that attitudes towards ICCDM were a significant predictor of behavioural intention, and behavioural intention and perceived behavioural control were identified as being significant predictors of ICCDM behaviour. However, subjective norms and perceived behavioural control did not predict the intention to engage in ICCDM behaviour in the current Sanming City sample.

The high score for collaborative attitudes showed that in the process of promoting the integration of the health service system, the health care personnel were aware of the importance of the interinstitutional collaboration as expected; thus, the ICCDM was highly recognized. This is consistent with the results of the qualitative study by Yuan et al. that primary health care professionals perceived vertical integration to result in improved professional competency, better care coordination and a stronger capacity to satisfy patients’ needs [11]. Another possible explanation could be that Sanming City is a model of medical reform in China; under the long-term influence of innovative thinking [20], health care personnel in Sanming usually have a positive attitude towards reform.

As mentioned above, the attitudes in this study positively affected behavioural intentions, which correspondingly positively affected ICCDM behaviour. Behavioural intention was identified as being the mediating variable of the transformation of behavioural attitudes into behaviours, which is consistent with TPB theory and other research on physician behaviours [21]. In other words, intention can serve not only as an internal driving process of attitude but also as a state of preparation for behaviour [22]. However, attitude was the only meaningful predictor of behavioural intention, thus indicating that ICCDM intention was derived more from personal perceptions and beliefs. This suggests that relevant departments should continue to strengthen policy publicity, continuously enhance health care personnel ‘s awareness of ICCDM and design training content according to the needs of primary health care providers to effectively improve the sense of gain in ICCDM, especially in other regions outside of Sanming. One recent study showed that the concept of vertical collaboration among primary health providers has not yet been formed, and collaboration still remains at the level of governance structure and business norms; thus, it is urgent to improve the internal identity of health care personnel for interinstitutional collaboration [23].

Surprisingly, our findings revealed that subjective norms and perceived behavioural control fail to explain ICCDM intention. Given the current lack of studies applying TPB in understanding ICCDM behaviour, it remains unclear as to whether subjective norms and perceived behavioural control are significant predictors of behaviour intention. When concerning subjective norms, one possible explanation is that Sanming’s integrated management of chronic diseases is a government action ; compared with the strong promotion of the government, whether colleagues and leaders support ICCDM is almost negligible. The same explanation applies to perceived behavioural control, wherein perceived barriers may not sway health care personnel’s ICCDM intentions. In short, further research is required to explore the potential factors that affect the association between perceived behavioural control and intention, as well as subjective norms and intention.

Although perceived behavioural control was not enough to influence intention in our study, it was an important predictor of ICCDM behaviour. Trafimow et al. divided perceived behavioural control into perceived abilities and perceived difficulties, and the results confirmed that such subdivisions could improve the explanatory ability of the TPB model [24]. Accordingly, we designed the measurement of perceived behavioural control, and the results showed that the score of perceived behavioural control was the lowest among all of the constructs. To some extent, this indicates that the ability of health care personnel to participate in ICCDM still needs to be strengthened, and they still perceive many practical obstacles.

Specifically, the three constructs with the lowest perceived control in this study were time and energy, experience and ability and guidelines. This is similar to China’s current reality. A recent study identified “heavy workloads” as being the biggest barrier to primary health care providers’ involvement in chronic disease management [25], and similar problems were observed in this sample. As the main force of the ICCDM, primary health care providers undertake not only public health work such as chronic disease management but also basic clinical services [26, 27]. Primary health care providers with multiple roles do not have the time or energy to juggle ICCDM, which may be the fundamental reason why they do not frequently engage in ICCDM behaviour, even within a strong intention. In addition, this study also suggests that the strengthening of the training related to collaboration, and the development of more detailed collaboration guidelines may promote more participation in ICCDM behaviour.

### Strengths, limitations and avenues for future research

The integration of the health service system is the focus of current medical reform in China and the world [28, 29]. This study applied TPB theory to analyse the collaborative behaviour of chronic disease management, which provided a new microscopic perspective for the current research on the integration of health service systems, and the relevant research results also further expanded the theoretical horizon of TPB. In addition, this study provided additional insights into which factors should be targeted to increase motivation and to promote ICCDM behaviour. Moreover, our findings may inform discussions in countries that have also integrated medical service through government orders.

This study also had several limitations that must be recognized. First, it depended on participants’ self-reported outcomes of ICCDM behaviour; thus, it may be at risk of a social desirability bias [30]. Second, as this study was conducted in Sanming City in China, the results should be interpreted with caution, as they were of limited generality. Third, although we used a well-researched and widely applied theory, it cannot be ruled out that we would have obtained factors other than TPB theory. In fact, subjective norms and perceived behavioural control did not explain cooperative intention in this study, thus suggesting that we may need to add other relevant factors to improve the explanatory power of the model. Future studies should further examine the influencing factors of ICCDM behaviour from a broader perspective to expand the existing TPB model and to better explain the ICCDM behaviour of health care personnel. For example, the perceived incentives of health care personnel could be considered in the model.

## Conclusions

This study may help researchers and policy-makers from other cities and countries to learn the current situation of integrated management of chronic diseases in Sanming City and the factors influencing health care personnel’s participation in ICCDM. In light of our results, and due to the fact that perceived behavioural control and attitudes and behavioural intention towards ICCDM behaviour were demonstrated to be significant predictors of ICCDM behaviour, these factors may be a promising focus of ICCDM interventions in the integrated management of chronic diseases in China. Furthermore, our findings implied that although health care personnel had strong collaborative attitudes and intentions, their self-reported collaborative behaviour was not frequent. There are many barriers between intention and behaviour, and a lack of time and energy is likely the most important reason. Moreover, our results also call for the need for system optimization, such as the further strengthening of education and training on how to effectively collaborate with chronic disease managers from other institutions and the further refinement of ICCDM guidelines and specifications.

## Electronic supplementary material

Below is the link to the electronic supplementary material.

Supplementary Material 1

## Data Availability

Anonymised data are available from the corresponding author upon reasonable request.

## Abbreviations

ICCDM: Interinstitutional Collaboration in Chronic Disease Management
TPB: Theory of Planned Behaviour
