# Supplementary material for: Explaining the intention and behaviours of interinstitutional collaboration in chronic disease management among health care personnel: a cross-sectional study from Fujian Province, China
Source: BMC Health Serv Res. 2023 May 11;23:477. doi: 10.1186/s12913-023-09453-0 (PMC10174609; doi:10.1186/s12913-023-09453-0)
Supplement: Supplementary file 1 — Supplementary Material 1 [file 12913_2023_9453_MOESM1_ESM.docx]

**Supplementary file 1: List of items measuring participants’ perception of ICCDM on TPB theory**

The following questions are intended to get your perception on " Interinstitutional Collaboration in Chronic Disease Management (ICCDM)". There are no right or wrong answers. Thank you very much!

| **Items** | Response (likert-5 scale) | References |
| --- | --- | --- |
| **Attitude of ICCDM** |  | Ajzen, I.  Francis, J.E.M., et al.  Yuan, S., et al.  LOU Ye, Z.Z.Q. |
| ICCDM is very important | Strongly disagree-Strongly agree  Strongly disagree-Strongly agree  Strongly disagree-Strongly agree  Strongly disagree-Strongly agree  Strongly disagree-Strongly agree |  |
| ICCDM helps improve service efficiency |  |  |
| ICCDM helps to save service costs |  |  |
| ICCDM leads to better quality of care for patients |  |  |
| ICCDM helps to improve the service capacity of medical staff |  |  |
| **Subjective Norms of ICCDM** |  | Ajzen, I.  Francis, J.E.M., et al.  Wang, D., et al. |
| Leadership attaches importance to ICCDM | Strongly disagree-Strongly agree |  |
| Colleagues are willing to contribute to ICCDM | Strongly disagree-Strongly agree |  |
| Medical staff from other institutions in the medical consortiums are actively involved in ICCDM | Strongly disagree-Strongly agree |  |
| **Perceived Behavior Control of ICCDM** |  | Ajzen, I.  Francis, J.E.M., et al.  Jin, Y., et al.  Zhang, M., et al. |
| I have the relevant experience and ability to participate in ICCDM | Strongly disagree-Strongly agree |  |
| I have enough time and energy to participate in ICCDM | Strongly disagree-Strongly agree |  |
| ICCDM has been supported by electronic information systems | Strongly disagree-Strongly agree |  |
| In the process of ICCDM, the business communication within the team is smooth | Strongly disagree-Strongly agree |  |
| There is a reasonable division of responsibilities between county (city) level hospitals and primary medical institutions in ICCDM | Strongly disagree-Strongly agree |  |
| There are reasonable guidelines and standards that can be followed in ICCDM | Strongly disagree-Strongly agree |  |
| **Behavior Intention of ICCDM** |  | Ajzen, I. |
| I am willing to participate in ICCDM | Strongly disagree-Strongly agree |  |
| ICCDM is the way I look forward to working in the future | Strongly disagree-Strongly agree |  |

Ajzen, I., Constructing a TPB Questionnaire: Conceptual and Methodological Considerations. 2002. <http://www-nix.oit.umass.edu/~ajzen/tpb.html>.

Francis, J. E. M., Johnston, M., Walker, A. E., Grimshaw, J. M., Foy, R., Kaner, E. F.

S., et al. (2004). “Constructing Questionnaires Based on the Theory of Planned

Behaviour: a Manual for Health Services Researchers,” in Newcastle upon Tyne:

Centre for Health Services Research (University of Newcastle upon Tyne).

Wang, D., et al., Applying Theory of Planned Behavior to Understand Physicians' Shared Decision-Making With Patients With Acute Respiratory Infections in Primary Care: A Cross-Sectional Study. Front Pharmacol, 2021. 12: p. 785419.<https://doi.org/10.3389/fphar.2021.785419>

Yuan, S., F. Fan and D. Zhu, Effects of Vertical Integration Reform on Primary Healthcare Institutions in China: Evidence From a Longitudinal Study. Int J Health Policy Manag, 2021. <https://doi.org/10.34172/ijhpm.2021.93>

LOU Ye, Z.Z.Q., Exploration of improving the enthusiasm of medical staff in public hospitals—taking Sanming health reform as example. Health Economics Research, 2016(9): p. 26-28. <https://doi.org/10.14055/j.cnki.33-1056/f.20160829.011>

Jin, Y., et al., Job satisfaction of the primary healthcare providers with expanded roles in the context of health service integration in rural China: a cross-sectional mixed methods study. Hum Resour Health, 2019. 17(1): p. 70. <https://doi.org/10.1186/s12960-019-0403-3>

Zhang, M., et al., Coping and compromise: a qualitative study of how primary health care providers respond to health reform in China. Hum Resour Health, 2017. 15(1): p. 50. <http://doi.org/10.1186/s12960-017-0226-z>
